# Notes and News

**Published:** 1893-08-12

**Authors:** 


### Notes and News.

Collected and Communicated.

There is a very strong feeling amongst the Governors of the London Hospital that a special meeting of the Governors must be held at once, or the interests of this great institution may be irretrievably and permanently injured. It is, we understand, proposed to present a requisition to the Committee in accordance with the bye-laws, but we hope the Committee will see their way to at once take the initiative, and call such a meeting without more loss of time. We further learn that the same sentiment prevails amongst the members of the medical staff, and that a majority of them are strongly of opinion that a special meeting of the Governors ought to be held forthwith.

The new hospital for the insane at Larbert was opened by Colonel Stirling, of Tarduf. The institution contains isolation wards for the special treatment of acute insanity, and will give Larbert Asylum an opportunity to test the efficacy of the new movement towards the adoption of clinical treatment for some phases of insanity. The occasion of the opening of such an addition to an asylum was an interesting one, and many connected with similar institutions were present.

During last month we have had a large amount of activity in the direction of new medical charities. Amongst others, the Cotswold Convalescent Home, on Cleeve Hill, four miles from Cheltenham, was opened by Sir J. Dorington, Bart., and owes its origin mainly to the generosity and energy of Mrs. Pitman, of Cheltenham. The new Scarborough Hospital was opened on the Royal wedding day. The Lady Hozier Convalescent Home at Lanark, the gift of Sir William Hozier to the Glasgow Western Infirmary, was opened by the Lord Provost of Glasgow. The foundation-stone of the new Lancaster Infirmary was laid by J. Williamson, Esq., M.P., and, lastly, the Corbett Hospital was opened at Brierley Hill.

We are glad to learn, in response to our remonstrance, that the managers of the Royal Infirmary, Edinburgh, have resolved to provide the additional accommodation required by the Triple Qualification Board for the clinical education of women.

The Pan-American Medical Congress are making great preparations for their excursion to Rome, to be present at the eleventh International Medical Congress in that city. The tour is to afford medical men excellent opportunities of becoming acquainted with various sanitary customs abroad.

The latest novelty in excursions—the cycling tour by members of the Royal Normal College for the Blind—came to an untimely end, as Dr. Campbell, the Founder and Principal, met with a serious accident through the breaking of the tandem on which he rode. Although the doctor is progressing favourably the rest of the party will not proceed with their tour.

The laboratory of the new buildings of the Chicago Medical College is now completed. It contains departments for physiology, physiological chemistry, pharmacy, chemistry, botany, pathology, histology, bacteriology, anatomy, and operative surgery, and has also lecture rooms and museums. Parallel with the establishment of the Medical College various hospitals are enlarging their capacity, so that suitable opportunities will be given in practical work to Chicago students.

The members of the St. John's Ambulance Brigade are under canvas at Muswell Hill for a week's training in ambulance drill, &c. The camp was opened by the Hon. Miss Amherst. There was an open competition for a handsome silver challenge cup, presented by the honorary surgeon, Dr. A. S. Eccles, in recognition of the good work done by the brigade on the occasion of the wedding of the Duke and Duchess of York.

The unusual prevalence of wasps this year is the subject of a good deal of newspaper correspondence, attention being principally directed to the destruction of the source of the evil—namely, the nests. We see now, however, that information is being asked as to the cure of the sting. The remedy nearest at hand is in most cases the most valuable, and we, therefore, advise our readers to try the immediate application of moist soil, and can testify that this will give prompt relief. Ammonia is a well known and excellent remedy, but it is not always attainable, and as nature mostly supplies an antidote, if we only have discovered it, the simplest and most available remedies are the best to rely on.

It is the fashion to steadily oppose the erection of a fever hospital in any locality, and then, fever occuring to abuse the authorities for their want of necessary provision. The sick are, perhaps, the worst sufferers from this course of action. At Salisbury the Local Government Board had forbidden the admission of small-pox into the workhouse, and no fever hospital was ready when a case occurred. The patient was dispatched from his dwelling on a fruitless journey to the workhouses in the district. He was taken back to his lodgings, whilst the authorities proceeded to borrow a tent for his reception. By the time it was erected he was too ill to be removed. Possibly his sojourn amongst the healthy may yet cause the erection of the tent to be not altogether in vain.

